# Probiotics: current evidences and new perspectives

**DOI:** 10.1186/1824-7288-40-S1-A46

**Published:** 2014-08-11

**Authors:** Valentina Fabiano, Chiara Mameli, Gian Vincenzo Zuccotti

**Affiliations:** 1 Università degli Studi di Milano, Department of Pediatrics, Luigi Sacco Hospital, 20157 Milan, Italy

## 

Probiotics are viable microorganisms that can exert potential benefits to human health. Use of probiotics in different human diseases and clinical conditions has gained much attention in the last decades. Several data exist about the potential benefits of probiotics in pediatric age for the management of some clinical conditions, with different and sometimes contrasting results. Currently, strong evidences support the use of probiotics for treatment of acute infectious diarrhoea and prevention of antibiotic-associated diarrhoea: bacteria belonging to the genus of lactobacilli and *Saccharomyces boulardii* showed the stronger evidences of benefits in different clinical trials and meta-analysis [1,2]. For other pediatric diseases and clinical conditions, evidences of the benefits of a probiotic supplementation are still not so strong. The recommendation to administer a probiotics supplementation in preterm, very low birth weight neonates at risk of developing necrotizing enterocolitis has recently been criticized by a critical review of the literature that conclude that well-designed clinical trials are still needed for suggesting its routinely use in these neonates [3]. Discussion is still ongoing about the benefits of probiotics in prevention or treatment of other pediatric diseases such as respiratory tract infections, urinary tract infections, Helicobacter pylori infection or allergic diseases. Clinical trials and meta-analysis about the use of probiotics in these pediatric clinical conditions have been published and still continued to be published; however, study designs are not always comparable and sometimes inadequate, species of probiotics used in the studies are different and the benefits demonstrated for one microorganism are not generalizable to other bacteria, results of the studies are still often not unequivocal or contrasting so that strong recommendations are still lacking. The use of probiotics has been suggested to be potentially beneficial for the management of gastrointestinal functional disorders, such as irritable bowel syndrome, again, with different results. These clinical conditions are supposed to be the result of a perturbation of the microbiota balance that leads to dysbiosis, responsible for the appearance of the characteristic symptoms. However, pathogenesis of gastrointestinal functional disorders appear to be much more complex and involve the nervous system and its bidirectional interactions with the intestine. Moreover, it has been hypothesized that intestinal microbial composition may itself influence end-points related to mood state, brain function, and mental outlook. A new fascinating perspective in studies about microbiota and probiotics is the characterization of the alterations of intestinal microbiome and their correlation with abnormalities in the bidirectional gut-brain interactions.
